# A Dynamic Integrated Fault Diagnosis Method for Power Transformers

**DOI:** 10.1155/2015/459268

**Published:** 2015-01-20

**Authors:** Wensheng Gao, Cuifen Bai, Tong Liu

**Affiliations:** ^1^Department of Electrical Engineering, Tsinghua University, Beijing 100084, China; ^2^State Grid Energy Research Institute, Beijing 102209, China; ^3^Electric Power Research Institute, CSG, Guangzhou 510080, China

## Abstract

In order to diagnose transformer fault efficiently and accurately, a dynamic integrated fault diagnosis method based on Bayesian network is proposed in this paper. First, an integrated fault diagnosis model is established based on the causal relationship among abnormal working conditions, failure modes, and failure symptoms of transformers, aimed at obtaining the most possible failure mode. And then considering the evidence input into the diagnosis model is gradually acquired and the fault diagnosis process in reality is multistep, a dynamic fault diagnosis mechanism is proposed based on the integrated fault diagnosis model. Different from the existing one-step diagnosis mechanism, it includes a multistep evidence-selection process, which gives the most effective diagnostic test to be performed in next step. Therefore, it can reduce unnecessary diagnostic tests and improve the accuracy and efficiency of diagnosis. Finally, the dynamic integrated fault diagnosis method is applied to actual cases, and the validity of this method is verified.

## 1. Introduction

Transformers play an essential role in power systems. Their unexpected failure may result in power blackouts and significant loss. To avoid the catastrophic result, dissolved gas analysis (DGA) is used widely to detect potential transformer failure at an early stage. Many effective diagnosis methods have been proposed based on DGA [1–11]. However, with DGA, only rough failure types (overheating or discharge) can be obtained. In order to get more detailed information about transformer condition, some researchers proposed various diagnosis models based on DGA and other diagnostic tests [12–15], which can get the detailed occurrence probability of each failure mode. These models are called integrated fault diagnosis models (IFDM) in this paper. IFDM can provide more information on transformer condition than the DGA method. Thus, it can serve transformer maintenance better and is significant to the power grid security control in smart grid.

The input parameters of IFDM are generally called evidence. Obviously, the more evidence is obtained, the more accurate the diagnosis result is. The most favorable situation is to know the status of all condition factors by means of monitoring or tests, but it is hard to realize due to the restriction of technical levels and economic conditions. Therefore, diagnostic tests should be performed selectively. Then, a related question arises: What are the selection rules? The primary objective is to reduce cost without loss of operation reliability. To achieve this aim, an optimized selection method is needed.

Existing integrated fault diagnosis methods do not involve this evidence-selection process; in other words, they focus on the diagnosis with obtained evidence in one step, no matter whether the evidence is valid or not. This diagnosis method is called static fault diagnosis mechanism (SFDM). SFDM does not optimize the evidence detection process. As a result, the evidence input into the diagnostic model may miss the most important one which is most related to transformer failure condition and cause an inaccurate diagnosis result. Some researchers briefly mentioned that the fault diagnosis of transformer should be a multistep process, but no specific embodiments and corresponding theoretical support were related [16–20]. As to this situation, this paper proposes a dynamic fault diagnosis mechanism (DFDM) based on the integrated fault diagnosis model, which comprises an evidence-selection process. It selects the evidence that better reflects the transformer condition so that unnecessary diagnostic tests can be reduced and the diagnosis accuracy can be improved. DFDM is of great significance to the developing smart grid.

The rest of this paper is organized as follows: Section 2 introduces the ideas of dynamic integrated fault diagnosis method.  Section 3 gives a brief introduction to Bayesian network, which is the basic theory of the model.  Section 4 reviews our previous research work.  Section 5 presents the detailed dynamic integrated fault diagnosis method. And then it is applied to two real cases in Section 6.  Section 7 is the conclusion part.

## 2. Ideas of Dynamic Integrated Fault Diagnosis Method

The dynamic integrated fault diagnosis method proposed in this paper means a dynamic fault diagnosis mechanism based on the integrated fault diagnosis model. Specifically speaking, our work can be divided into two parts. One is to improve the existing IFDM, and the other is to propose a dynamic fault diagnosis mechanism based on the improved IFDM.

Referring to IFDM, obviously, the more failure-related information is considered, the more accurate the diagnosis result is. The information related to transformer failure condition can be primarily divided into two types: influence factors and characterization factors. Influence factors are the factors that may cause transformer failure, such as abnormal working conditions and family defects. Characterization factors mean the failure symptoms detected by means of various diagnostic tests, such as DGA, partial discharge test. Because both influence factors and characterization factors are diverse and the relationship between factors and failure modes is difficult to quantitatively describe, establishing a complete and accurate integrated fault diagnosis model is a tough task. The relationship between characterization factors and failure modes is relatively easier to obtain. In existing integrated fault diagnosis models, only characterization factors are considered [12–14]. However, lack of consideration of influence factors may cause inaccurate diagnosis result because they affect transformer failure condition directly and can significantly increase the occurrence probability of certain failure modes. For example, the experience of external short circuit often corresponds to winding deformation. Therefore, it is necessary to add influence factors to the existing fault diagnosis model to get more accurate result. This is the first work which has been done in this paper.

More importantly, the main purpose of this paper is to propose a dynamic fault diagnosis mechanism based on the improved IFDM. The building process of DFDM is directly related to the diagnostic technique used in IFDM. IFDM can be established by means of analyzing the relationship between transformer failure modes, its influence factors and characterization factors. However, the failure process of transformer is complex, and its influence factors and characterization factors are diverse, fuzzy, and incomplete. The relationship between factors and failure modes is not one to one. As to the characteristics mentioned, existing studies on the relationship between failure modes and characterization factors generally use intelligent diagnostic techniques, such as neural networks, Bayesian network (BN), expert systems, and evidential reasoning methods [12–14]. In these intelligent diagnostic methods, Bayesian network is a suitable technique for IFDM because it is a probabilistic causal network, and the relationship between influence factors and failure modes or failure modes and characterization factors is just probabilistic causal. Influence factors, failure modes, and characterization factors can be intuitively described by a three-layer BN.

Thus, BN is considered as the basic theory of the dynamic integrated fault diagnosis method proposed in this paper. It is necessary to emphasize the difference of the method proposed in this paper since BN has been used in transformer fault diagnosis. On one hand, most of existing transformer diagnosis models using BN are established based on DGA data. Few have taken the diagnostic tests into account. In other words, few studies have been done on the integrated fault diagnosis model summarized in Section 1. Furthermore, the transformer fault diagnosis model considering both characterization factors and influence factors has not been reported yet, and this work will be done in next section. On the other hand, as mentioned in the previous section, all the existing fault diagnosis models of transformers based on BN are SFDM, without the evidence-selection process. The DFDM will be proposed in Section 5.

BN is a powerful tool in transformer fault diagnosis and is the basic algorithm of DFDM. A brief introduction to it will be given in next section.

## 3. A Brief Introduction to Bayesian Network

**Figure 1:**
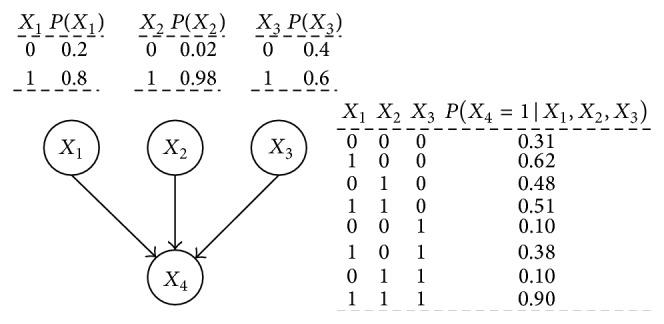
A diagram of Bayesian network.

Bayesian network includes two parts: network structure and network parameters. Network structure is the qualitative part of BN, while network parameters are the quantitative part. The structure of BN is described with a directed acyclic graph, as shown in Figure 1. *X*_1_, *X*_2_, *X*_3_, and *X*_4_ represent random variables, where *X*_1_, *X*_2_, and *X*_3_ are root nodes and *X*_4_ is a child node. The arcs between nodes describe the condition dependence between random variables. Network parameters mean each node in the network structure has a conditional probability table (CPT). As shown in Figure 1, the CPTs of root nodes *X*_1_, *X*_2_, and *X*_3_ are their marginal probability distributions *P*(*X*_1_), *P*(*X*_2_), and *P*(*X*_3_), and the CPT of the child node *X*_4_ is its conditional probability distribution *P*(*X*_4_∣*X*_1_, *X*_2_, *X*_3_). With the specific conditional independence of BN, the joint probability distribution can be simplified as [21]

(1) PX1,X2,X3,X4 =∏i=14PXi ∣ πXi =PX1PX2PX3PX4 ∣ X1,X2,X3,

wherein *π*(*X*_*i*_) represents the parent node of *X*_*i*_  (*i* = 1,2, 3,4).

**Figure 2:**
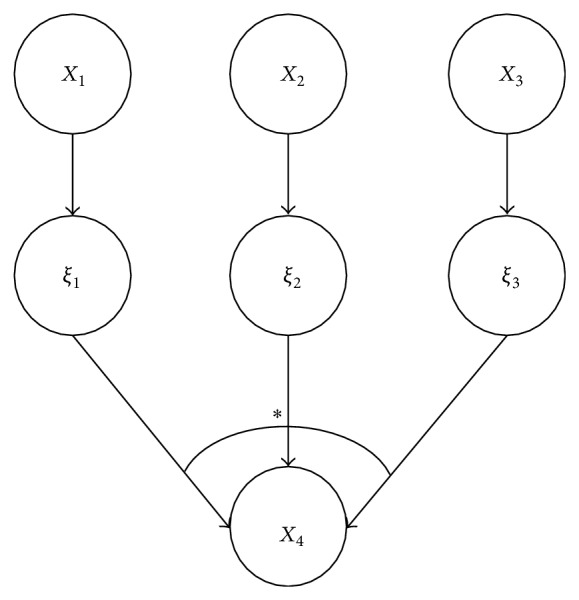
A diagram of independent influence.

**Figure 3:**
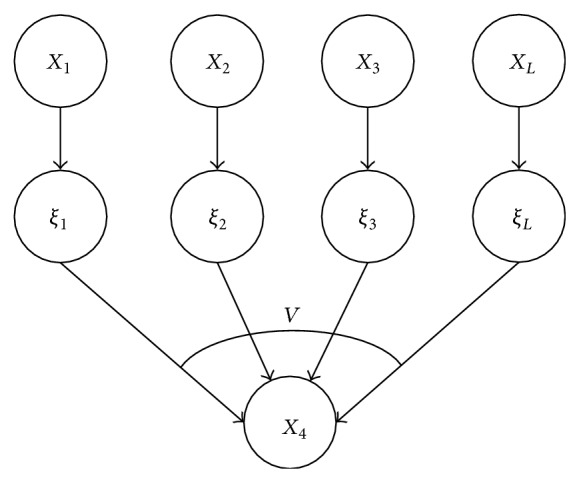
A diagram of leaky noisy-or node.

In Figure 1, the random variables are binary (0 or 1). Thus, eight independent parameters are needed for the CPT of node *X*_4_, and it is difficult if there is not enough data. In practice, random variables may have more states. It means more parameters are required to get the CPT, and therefore the difficulty increases. In this case, it is often assumed that the impact of each parent node on the child node is independent. If *X*_1_, *X*_2_, and *X*_3_ independently affect *X*_4_, then for any *α* ∈ *Ω*_*X*_4__ (*Ω*_*X*_4__ denotes the state space of *X*_4_), the following relationship exits: [21] (as shown in Figure 2):

(2) PX4=α ∣ X1,X2,X3 =∑α1∗α2∗α3=αPξ1=α1 ∣ X1Pξ2=α2 ∣ X2     ×Pξ3=α3 ∣ X3,

where “∗” is a basic composite operator, *ξ*_*i*_ is the contribution of *X*_*i*_ to *X*_4_, and *P*(*ξ*_*i*_∣*X*_*i*_) is the contribution probability distribution of *X*_*i*_ to *X*_4_  (*i* = 1,2, 3). When the basic composition operator is “logical or,” the node *X*_4_ is noisy-or node; when it is “logical and,” *X*_4_ is noisy-and node. Under the assumption of independent influence, the conditional probability distribution *P*(*X*_4_ = *α*∣*X*_1_, *X*_2_, *X*_3_) can be obtained from the contribution probability distribution of *X*_1_, *X*_2_, and *X*_3_, and the number of necessary parameters reduces greatly. Additionally, not all the causes of a child node can be considered in its parent nodes in a BN, and therefore a leaky node is usually used to represent the causes not considered. For example, a leaky noisy-or node represents when the values of all parent nodes are 0, its value is still possible to be 1 [22]. As shown in Figure 3, *X*_*L*_ represents a leaky node, and “*v*” represents the synthesis operator is “or.” Leaky noisy-or node is a widely used method to simplify parameters in Bayesian network.

The establishment of a Bayesian network first needs to determine the study objects, namely, the random variables in the network. And then based on the analysis of the causal relationship between random variables, build the network structure. Finally, introduce the network parameters by the network structure. After the establishment of a Bayesian network, many algorithms can be selected to do the diagnosis. Joint tree algorithm is a commonly used inference method, and there is much mature software such as MATLAB toolbox BNT. The details of calculation process can be found in [21].

## 4. Review of Our Previous Work

**Figure 4:**
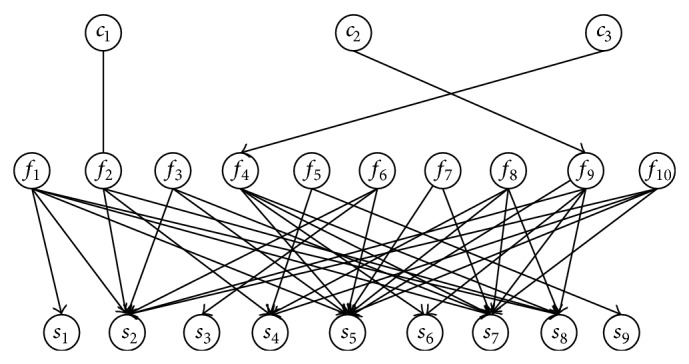
The integrated diagnosis model in our previous research work [15].

A three-layer BN model involving 10 common failure modes, 3 abnormal working conditions, and 9 failure symptoms detected from diagnostic tests has been established in our previous research work [15], as shown in Figure 4 and Table 1. The failure modes and failure symptoms considered in this model are common, and their relationship is also discussed in some papers [12–14], but no influence factors have been considered into the model yet. The main contribution of our previous research work [15] is to add the influence factors to the diagnosis model, and the validity of our model has been verified. Compared with the existing models without influence factors, our model usually can get a more accurate diagnosis result.

In Figure 4, abnormal working conditions, failure modes, and abnormal symptoms are represented by nodes, and their causal relationship is represented by directed arcs. The graphical representation expresses the conditional independence relationship between nodes, and the conditional independent relationship decreases the parameters needed for total probability. If all child nodes are considered as leaky noisy-or nodes, only the prior probability of parent nodes and the contribution probability of parent node to child nodes are needed. The probability parameters are acquired from transformer failure statistics and empirical approach. The rest of the details can be found in [15].

The previous model also has some limitations, and it is improved in the following sections.

## 5. Dynamic Integrated Fault Diagnosis Method

### 5.1. Improvement of the Previous Integrated Fault Diagnosis Model

It should be noted that only 3 abnormal working conditions are considered in the model shown in Figure 4, and other influence factors are missed. There are two reasons. On one hand, factors causing transformer failure are many, and some failure theory is not clear enough to consider them into the model reasonably. According to the transformer failure statistics [23–25], lots of transformer failures result from abnormal working conditions, which primarily includes abnormal overload, external short circuit, and lightening. Therefore, these three types of abnormal working conditions are included in the model. On the other hand, even though the other influence factors are not considered in the model, their damage to the transformer can be effectively detected through certain diagnostic techniques. From this point of view, missing them may not bring much error. Referring to abnormal working conditions, the damage caused by them generally has accumulated effect. The failure symptoms detected by diagnostic techniques may not be able to accurately reflect the real condition of transformers. In other words, it is difficult to measure and distinguish different damage levels caused by different times of abnormal working conditions that transformers are suffered. Hence, it is necessary to incorporate them in the model. Based on the two points, only abnormal overload, external short circuit, and lightening seem as the influence factors in the IFDM.

**Figure 5:**
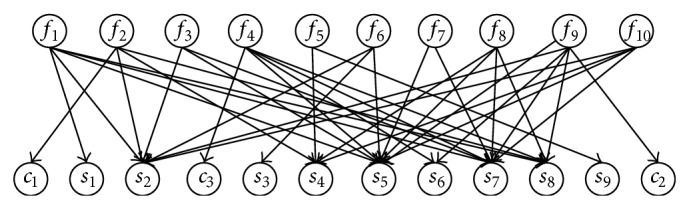
Integrated fault diagnosis model proposed in this paper.

The model in Figure 4 intuitively describes the causal relationship between abnormal working conditions, failure modes, and failure symptoms. However in [15], the model is more accurate compared with the existing model without influence factors. It is found that sometimes the direct application of this model may bring diagnostic wrong result due to the incomplete nodes. As to this limitation, this paper improves the model, as shown in Figure 5. Abnormal working conditions are changed into a special type of characterization factors, and they are supporting evidence information of failure symptoms. Thus, the error due to lack of influence factors is improved, and also the effect of abnormal working condition on transformer is considered. The probability relationship in Figure 5 is obtained based on our previous work [15] and fault statistics of CIGRE [25], as Table 2 shows.

The data in Table 2 means the probability of the occurrence of each failure symptom or the experience of each abnormal working condition when a transformer is suffering from a failure mode. These values are obtained based on a lot of transformer failure data and experts' experience and are used to present DFDM method. The fault statistics of CIGRE is chosen as a data resource because it is a global and comprehensive investigation. It should be noted that the values of Table 2 are variable since they primarily depend on the fault data source, and they will be more accurate if deducing them from the latest transformer fault data. Furthermore, if the fault data that we are able to collect in future is enough to be categorized into rated power, voltage level, technology, and so forth with statistical significance, it will be more invaluable, because the characteristics of a transformer will affect the probability rates of Table 2.

After the integrated diagnosis model is established, given the existing evidence, it can compute the posterior probability of each failure mode. Generally, the failure mode with the maximum probability is considered the one most likely to occur. This inference rule is called maximum a posteriori probability (MAP) estimation. MAP estimation is the main estimation method in DFDM presented in next section.

### 5.2. Dynamic Fault Diagnosis Mechanism

In this section, a dynamic fault diagnosis mechanism is presented to optimize the actual process of fault diagnosis. In reality, evidence information is generally acquired through different means of detection, and therefore the evidence acquisition is a continuous updating process. With the evidence updated, fault diagnosis result is also constantly corrected, gradually approaching the true condition of transformers. This gradual correction process of fault diagnosis is called dynamic fault diagnosis process. DFDM refers to the theoretical basis at each step in this process, mainly including two aspects: diagnosing the most possible failure mode in current step and determining the evidence information needed in next step. The first aspect is the traditional static fault diagnosis mechanism. It can be seen that, compared to the static fault diagnosis mechanism, a dynamic mechanism adds the part of estimating the evidence information needed. This key part can reduce the blindness of fault diagnosis process and diagnose transformer condition more efficiently.

A generalized transformer fault diagnosis process includes abnormality detection, fault diagnosis through tests, and the final check by hanging core. From an economic point of view, the most easily accessible evidence should be collected firstly, usually including online monitoring information, operating experience, familial defective information, and historical maintenance records. Then, based on the information, preliminarily determine whether the transformer is abnormal and whether offline diagnostic tests are needed. If needed, priority should be given to live tests without outage. Based on the test result, decide whether to implement outage tests. Finally, perform the internal check by hanging core, founding the true failure condition. Dynamic fault diagnosis process in this paper includes all the steps before the final one, and the hanging core inspection is considered as a method to verify the diagnosis result.

Based on the obtained priority of evidence discussed above, the evidence of the integrated model in Figure 5 is divided into three layers, as shown in Table 3. The evidence in the first layer is most easily accessible, including abnormal working conditions, historical maintenance records, and online monitoring information. The second layer includes live tests, and outage tests compose the third layer. It is worth mentioning that, in general, online monitoring device has not yet been widely used. In order to facilitate the description of DFDM in this paper, an assumption is made that DGA is an online monitoring approach, and the others are offline monitoring methods.

Based on the integrated fault diagnosis model and evidence division, DFDM is developed. As mentioned above, DFDM can be attributed to two problems: (1) find the most possible failure mode based on the existing evidence in current step; (2) if the occurrence of this failure mode is not fully determined, propose the diagnostic test to be performed in next step. These two problems can be typically solved by the MAP inference of Bayesian network. Accordingly, the full process of dynamic integrated fault diagnosis method is presented as follows:

1. Regard all available evidence in the first layer in Table 3 as *E*.
2. Put evidence *E* into the integrated fault diagnosis model in Figure 5 to find the failure mode *f*_*i*_ with the maximum probability *P*. If *P* > *P*_set_ (*P*_set_ is a probability threshold previously set), then go to (5); else go to (3).
3. Assume *f*_*i*_ has occurred; put [*E*, *f*_*i*_] into the integrated fault diagnosis model in Figure 5 to find the diagnostic test *s*_*i*_ with the maximum probability (the diagnostic test obtained priority in Table 3 should also be considered). Then, *s*_*i*_ is the test that should be performed in next step.
4. Perform test *s*_*i*_ to find whether there is abnormal or not, and add the result into evidence *E* = [*E*, *s*_*i*_] (*s*_*i*_ means it is abnormal), or $E=[E,\overset{-}{s_{i}}]$ ($\overset{-}{s_{i}}$ means it is not abnormal). Then go to (2).
5. *f*_*i*_ is the final failure mode.

## 6. Case Study

In this section, the dynamic integrated fault diagnosis method is applied to two real cases. Case 1 refers to a diagnosis problem only by means of diagnostic tests. In Case 2, the transformer has experienced abnormal working conditions. Thus, both abnormal working conditions and diagnostic tests are treated as evidence to diagnose the transformer failure condition. In order to better indicate the benefits of DFDM, both DFDM and traditional SFDM are applied, and a comparison between the results is made.

### 6.1. Case 1

Case 1 can be described as follows [14]: the oil chromatographic analysis results of a transformer indicated an overheating abnormal symptom; the tests results of water content in transformer oil, partial discharge and earthing current of core, *φ*(co⁡)/*φ*(co⁡_2_) were all normal.

Based on the known conditions, the evidence can be described as $E=(s_{2},\overset{-}{s_{1}},\overset{-}{s_{4}},\overset{-}{s_{5}},\overset{-}{s_{7}},\overset{-}{s_{8}})$. In the following, diagnosis processes based on SFDM and DFDM are analyzed.

*Static Fault Diagnosis Mechanism.* In this case, the evidence without selection is $E=(s_{2},\overset{-}{s_{1}},\overset{-}{s_{4}},\overset{-}{s_{5}},\overset{-}{s_{7}},\overset{-}{s_{8}})$. Put the evidence directly into the model in Figure 5, and the posterior probability of each failure mode is obtained, as shown in Table 4.

It can be seen that failure mode *f*_6_ is with the maximum probability, so the diagnosis result is failure of tap-changer. It agrees well with the hanging core inspection result.

*Dynamic Fault Diagnosis Mechanism.* The diagnosis process of DFDM is described as follows (the probability threshold *P*_set_ is assumed to be 0.8).

**Step 1 .**

The available evidence in the first layer $E=(s_{2},\overset{-}{s_{5}})$.

**Step 2 .**

Put the evidence *E* into the model in Figure 5 to find the most possible failure mode *f*_1_ with probability *P* = 0.1965 < *P*_set_.

**Step 3 .**

Assume *f*_1_ has occurred; put [*E*, *f*_1_] into the model in Figure 5 to obtain the diagnostic test *s*_1_ with the maximum probability.

**Step 4 .**

Perform the test of *s*_1_, and the result shows it is normal; then $E=(s_{2},\overset{-}{s_{5}},\overset{-}{s_{1}})$.

**Step 5 .**

Put the evidence *E* into the model in Figure 5 to find the most possible failure mode *f*_6_ with probability *P* = 0.1081 < *P*_set_.

**Step 6 .**

Assume *f*_6_ has occurred; put [*E*, *f*_6_] into the model in Figure 5 to obtain the diagnostic test *s*_3_ with the maximum probability.

**Step 7 .**

Put the evidence *E* into the model in Figure 5 to find the most possible failure mode *f*_6_ with probability *P* = 0.9134 > *P*_set_. Accordingly, the diagnosis process ends, and the diagnosis result is also *f*_6_ failure of tap-changer.

The comparison between SFDM and DFDM is listed in Table 5. It can be seen that, in SFDM, 6 types of evidence are needed to perform the diagnosis, and DFDM only needs 4. Additionally, though with less evidence, DFDM can get a more reliable diagnosis result compared to SFDM (posterior probability: 0.9134 versus 0.1147). It can be seen that DFDM can provide the most possible failure mode and the most effective diagnostic test should be done in next step (three-phase unbalanced factor of winding DC resistance (*s*_3_)), which makes the diagnosis process more directional and effective.

### 6.2. Case 2

Case 2 refers to a transformer that experiences abnormal working conditions. Thus, both abnormal working conditions and diagnostic test are treated as evidence to diagnose transformer failure condition.

This case can be described as follows: a transformer did not experience external short circuit, and the arrester has acted once. Its oil chromatographic analysis results are shown in Table 6, and absorption ratio and polarization index, three-phase unbalanced factor of winding DC resistance, and deviation of winding ratio were all within normal range.

According to Table 6, the three-ratio-code of this transformer is 102, that is, discharge abnormal symptom. Therefore, the evidence is known as $E=(c_{3},s_{5},\overset{-}{c_{2}},\overset{-}{s_{2}},\overset{-}{s_{3}},\overset{-}{s_{6}},\overset{-}{s_{8}},\overset{-}{s_{9}})$. In the following, diagnosis processes based on SFDM and DFDM are analyzed.

*Static Fault Diagnosis Mechanism.* In this case, the evidence without selection is $E=(c_{3},s_{5},\overset{-}{c_{2}},\overset{-}{s_{2}},\overset{-}{s_{3}},\overset{-}{s_{6}},\overset{-}{s_{8}},\overset{-}{s_{9}})$. Put the evidence directly into the model in Figure 5, and the posterior probability of each failure mode is obtained, as shown in Table 7.

It can be seen that failure mode *f*_4_ is with the maximum probability, so the diagnosis result is winding short circuited. The hanging core inspection found its insulation in medium-voltage side is damaged and short circuited, which agrees well with the diagnosis result.

*Dynamic Fault Diagnosis Mechanism.* The diagnosis process of DFDM is described as follows (the probability threshold *P*_set_ is assumed to be 0.8).

**Step 1 .**

The available evidence in the first layer $E=(c_{3},s_{5},\overset{-}{c_{2}},\overset{-}{s_{2}})$.

**Step 2 .**

Put the evidence *E* into the model in Figure 5 to find the most possible failure mode *f*_4_ with probability *P* = 0.6879 < *P*_set_.

**Step 3 .**

Assume *f*_4_ has occurred; put [*E*, *f*_4_] into the model in Figure 5 to obtain the diagnostic test *s*_7_ with the maximum probability.

**Step 4 .**

Perform the test of *s*_7_, and the result shows an abnormal symptom exists; then $E=(c_{3},s_{5},s_{7},\overset{-}{c_{2}},\overset{-}{s_{2}})$.

**Step 5 .**

Put the evidence *E* into the model in Figure 5 to find the most possible failure mode *f*_4_ with probability *P* = 0.8603 > *P*_set_. Accordingly, the diagnosis process ends, and the diagnosis result is also *f*_4_ winding short circuited.

The comparison between SFDM and DFDM is listed in Table 8. From Table 8, the same conclusion as Case 1 can be drawn.

## 7. Conclusion

A dynamic integrated fault diagnosis method based on Bayesian network is proposed in this paper. Different from the existing static fault diagnosis mechanism, it is a step by step method. It can provide the most possible failure mode and the most effective diagnostic test should be done in next step. Therefore, it can reduce unnecessary diagnostic tests and improve the accuracy and efficiency of diagnosis.

## Figures and Tables

**Table 1:** Node description in Figure 4 [15].

Node description (symbol)	
Abnormal working conditions (*c*)	Abnormal overload (*c*_1_) External short circuit (*c*_2_) Lightening (*c*_3_)

Failure symptoms (*s*)	Earthing current of core (*s*_1_) An overheating abnormal symptom indicated by three-ratio-code (*s*_2_) Three-phase unbalanced factor of winding DC resistance (*s*_3_) Water content in transformer oil (*s*_4_) A discharge abnormal symptom indicated by three-ratio-code (*s*_5_) Deviation of winding ratio (*s*_6_) Partial discharge (*s*_7_) *φ*(co)/*φ*(co_2_)(*s*_8_) Absorption ratio or polarization index (*s*_9_)

Failure modes (*f*)	Multipoint earthing of core (*f*_1_) Insulation aging (*f*_2_) Overheating with flux leakage (*f*_3_) Winding short circuited (*f*_4_) Insulation dampened (*f*_5_) Failure of tap-changer (*f*_6_) Suspended discharge (*f*_7_) Discharge in barrier (*f*_8_) Winding deformation (*f*_9_) Discharge in transformer oil (*f*_10_)

**Table 2:** Parameters of integrated fault diagnosis model.

Failure mode (prior probability (×10^−2^))	*s*_1_	*s*_2_	*s*_3_	*s*_4_	*s*_5_	*s*_6_	*s*_7_	*s*_8_	*s*_9_	*c*_1_	*c*_2_	*c*_3_
*f*_1_(0.45)	0.9	0.82			0.19		0.3	0.2				
*f*_2_(0.11)		0.22		0.27				0.82		0.02		
*f*_3_(0.13)		0.71			0.29		0.35					
*f*_4_(0.12)					0.52	0.8	0.9	0.68				0.55
*f*_5_(0.10)				0.72					0.75			
*f*_6_(0.26)		0.67	0.87		0.23							
*f*_7_(0.16)					0.86		0.9					
*f*_8_(0.28)				0.42	0.88		0.9	0.76				
*f*_9_(0.24)		0.15			0.68	0.8	0.75	0.72			0.24	
*f*_10_(0.14)		0.2		0.6	0.7		0.9					
Leaky node	0.01	0.01	0.01	0.01	0.01	0.01	0.01	0.01	0.01	0.01	0.01	0.01

**Table 3:** Evidence division from an economic point of view.

Layer	Types of evidence	Diagnosis technologies
1	Abnormal working conditions	c_1_, c_2_, c_3_
	Online monitoring information	s_2_, s_5_
	Historical maintenance records	Historical information (c_1_~c_3_, s_1_~s_9_)

2	Live tests	s_1_, s_4_, s_7_, s_8_

3	Outage tests	s_3_, s_6_, s_9_

**Table 4:** Diagnosis result of static fault diagnosis mechanism in Case 1.

Symbol of failure mode	Probability of occurrence	Symbol of failure mode	Probability of occurrence
*f*_1_	0.0142	**f**_6_	**0.1147**
*f*_2_	0.0027	*f*_7_	0
*f*_3_	0.0348	*f*_8_	0
*f*_4_	0	*f*_9_	0.0007
*f*_5_	0.0003	*f*_10_	0.0003

**Table 5:** Diagnosis process comparison between SFDM and DFDM in Case 1.

	SFDM	DFDM
Evidence amount	6	4
Diagnosis result		
Failure mode	*f*_6_	*f*_6_
Posterior probability	0.1147	0.9134

**Table 6:** DGA result.

Gas	CH_4_	C_2_H_4_	C_2_H_6_	C_2_H_2_	H_2_	CO	CO_2_
Content (*μ*L/L)	6.99	10.87	0	3.72	13.57	229	2344

**Table 7:** Diagnosis result of static fault diagnosis mechanism in Case 2.

Symbol of failure mode	Probability of occurrence	Symbol of failure mode	Probability of occurrence
*f*_1_	0.0088	*f*_6_	0.0018
*f*_2_	0.0002	*f*_7_	0.0905
*f*_3_	0.0071	*f*_8_	0.0396
**f**_4_	**0.1475**	*f*_9_	0.0039
*f*_5_	0.0003	*f*_10_	0.0530

**Table 8:** Diagnosis process comparison between SFDM and DFDM in Case 2.

	SFDM	DFDM
Evidence amount	8	5
Diagnosis result		
Failure mode	*f*_4_	*f*_4_
Posterior probability	0.1475	0.8603

## References

[1] Ibrahim M. M., Sayed M. M., Abu El-Zahab E. E. Diagnosis of power transformer incipient faults using fuzzy logic-IEC based approach.

[2] Zhao A., Tang X., Zhang Z., Liu J.-H. The DGA interpretation method using relative content of characteristic gases and gas-ratio combinations for fault diagnosis of oil-immersed power transformers.

[3] van Le N. Application of artificial intelligence in diagnosis of power transformer incipient faults.

[4] Huang Y.-C., Sun H.-C. (2013). Dissolved gas analysis of mineral oil for power transformer fault diagnosis using fuzzy logic. *IEEE Transactions on Dielectrics and Electrical Insulation*.

[5] Ma D., Zhang W., Yao W. Establish expert system of transformer fault diagnosis based on dissolved gas in oil.

[6] Zhou A. H., Yi Y., Hong S., Zeng X. H. Power transformer fault diagnosis based on integreted of rough set theory and evidence theory.

[7] Ahmed M. R., Geliel M. A., Khalil A. Power transformer fault diagnosis using fuzzy logic technique based on dissolved gas analysis.

[8] Wang X., Li Q., Li C., Yang R., Su Q. (2013). Reliability assessment of the fault diagnosis methodologies for transformers and a new diagnostic scheme based on fault info integration. *IEEE Transactions on Dielectrics and Electrical Insulation*.

[9] Lv H. Study on fault diagnosis of power transformer based on RSNN.

[10] Souahlia S., Bacha K., Chaari A. SVM-based decision for power transformers fault diagnosis using Rogers and Doernenburg ratios DGA.

[11] Xie Q. J., Zeng H. X., Ruan L., Chen X. M., Zhang H. L. Transformer fault diagnosis based on bayesian network and rough set reduction theory.

[12] Velasquez-Contreras J. L., Sanz-Bobi M. A., Galceran Arellano S. (2011). General asset management model in the context of an electric utility: application to power transformers. *Electric Power Systems Research*.

[13] Su H. S. Transformer fault diagnosis method based on rough set and bayesian optimal classifier.

[14] Wang Y. Q., Lu F. C., Li H. M. (2008). Synthetic fault diagnosis method of power transformer based on rough set theory and bayesian network. *Advances in Neural Networks—ISNN 2008*.

[15] Bai C. F., Gao W. S., Jin L., Yu W. X., Zhu W. J. (2013). Integrated diagnosis of transformer faults based on three-layer Bayesian network. *High Voltage Engineering*.

[16] CIGRE WG A2.20 Guide on economics of transformer management.

[17] Gray I. Condition-based strategies for transformer age assessment. https://www.google.com.hk/url?sa=t&rct=j&q=&esrc=s&source=web&cd=1&ved=0CC8QFjAA&url=http%3a%2f%2fsatcs%2eco%2eza%2fTransformer_Age_Assessment%2epdf&ei=LZJoUsK_M62kigfh-IDgCw&usg=AFQjCNHvgCnImByvFoXzTnSH9E2PR7j6kQ.

[18] Sokolov V. V. Considerations on power transformer condition-based maintenance.

[19] Arshad M., Islam S. M. Power transformer condition monitoring and assessment for strategic benefit. https://www.google.com/url?url=http://scholar.google.com/scholar_url%3Furl%3Dhttp://metalab.uniten.edu.my/~farrukh/vcb/Vaccuminterrup/Transformer%252520Condition%252520Monitoring/Arshad2003.pdf%26hl%3Dzh-CN%26sa%3DX%26scisig%3DAAGBfm3_3Hdl5Wks1vsHOKSxGdxHHULJ1g%26nossl%3D1%26oi%3Dscholarr&rct=j&q=&esrc=s&sa=X&ei=AdWzVJ-rGMqpogSe54C4CQ&ved=0CB0QgAMoADAA&usg=AFQjCNFvrC_B6ezY6qFazP7I6HJDa7_4UA.

[20] Setayeshmehr A., Akbari A., Borsi H., Gockenbach E. A procedure for diagnosis and condition based maintenance for power transformers.

[21] Zhang L., Guo H. (2006). *Introduction of Bayesian Network*.

[22] Zhou S. (2010). *Study on the Fault Diagnosis Method of Power System Based on Bayesian Network*.

[23] Wang M., Vandermaar A. J., Srivastava K. D. (2002). Review of condition assessment of power transformers in service. *IEEE Electrical Insulation Magazine*.

[24] Franzen A., Bertling L. State of the art-life time modeling and management of transformers. http://www.foxgo.net/uploads/2/1/3/8/2138775/foxgo-life_time_modelling_of_transformers.pdf.

[25] CIGRE (1983). An international survey on faults in large power transformers in service. *Electra*.

